# Tariffs, transportation, and profits in cross-border e-commerce: A dual-channel supply chain decision-making strategy

**DOI:** 10.1371/journal.pone.0309535

**Published:** 2025-01-08

**Authors:** Yihang Guo, Xianle Wang, Zhongwei Yang, Kaimin Chen, Wei Weng

**Affiliations:** 1 School of Economics and Management, Yunnan Normal University, Kunming, China; 2 International School of Technical Education, Sichuan College of Architertural Technology, Deyang, China; 3 Taylor’s University, Subang Jaya, Malaysia; Harbin Institute of Technology, CHINA

## Abstract

The development of cross-border e-commerce platform promotes the new channel model between domestic and international. How to determine the dual-channel pricing decision of manufacturers and retailers under the condition of tariff and transportation heterogeneity has become an important and realistic problem. Based on the perspective of cross-border e-commerce dual-channel supply chain, this paper considers the impact of import tariff, transport heterogeneity and export tax rebate, compares and analyzes the performance difference between decentralized decision-making and centralized decision-making, and analyzes the impact of import tariff, export tax rebate and transport heterogeneity on cross-border e-commerce dual-channel pricing, demand and profit. The results show that the tariff is positively correlated with the manufacturer’s direct selling price and the retailer’s retail price, while the tariff is negatively correlated with the wholesale price, the demand and profit of direct selling channel and the retail channel. Export tax rebate rate is positively correlated with manufacturers’ demand and profit and retailers’ demand and profit, and negatively correlated with manufacturers’ wholesale price, direct selling price and retail price. The increase of unit freight in direct channel is unfavorable to manufacturers and beneficial to retailers; The increase in unit freight rates in retail channels is bad for both manufacturers and retailers. Centralized decision-making is beneficial to supply chain demand and profits, and can improve the overall performance of the supply chain.

## 1. Introduction

The development of cross-border e-commerce platforms provides a new online channel for local products to explore the international market, and more and more local manufacturers not only begin to consolidate and expand traditional sales channels, but also start to sell overseas through cross-border e-commerce platforms and transport products to overseas consumers [1]. Therefore, cross-border e-commerce has become an important way for local manufacturers to enhance their competitiveness and performance in the global market [2]. However, the current market competition among multinational enterprises is intensifying, and tariffs and transportation costs occupy a key position in the development of multinational enterprises. Statistics show that since March 2018, the trade friction between China and the United States has continued to escalate, and the two sides have experienced four rounds of tariff increases, which directly leads to a significant increase in the cost of cross-border product transactions [3], further affecting consumers’ purchase decisions, and thus making cross-border supply chains face unusually severe challenges.

Moreover, cross-border e-commerce has changed the distribution model of local manufacturers [4]. On the one hand, manufacturers still wholesale products to downstream overseas retailers, and then retailers sell them to customers at their own prices [5]. On the other hand, manufacturers will also build their own online direct sales channels (self-operated flagship stores) on cross-border e-commerce platforms (Dunhuang, Taobao, Amazon) to sell directly to overseas customers [6]. This means that cross-border e-commerce has created a dual-channel structure for local manufacturers, which may bring both opportunities and challenges for their performance [7].

According to existing studies, there are many challenges in the development of dual-channel supply chain [8]. However, the main problem related to this paper focus on two aspects. The first is the balance and coordination of cross-border supply chain; The other is the strategy adjustment of dual-channel supply chain considering the competition of manufacturers and markets. In the dual-channel supply chain, traditional channels and network channels are always in a competitive state, and different pricing will determine different purchasing decisions of consumers. For example, low pricing of network channels will easily induce consumers to switch from traditional channels to online channels, thus reducing the demand for traditional retailers [9]. This reduces revenue and profit levels throughout the supply chain. Therefore, based on the background of channel coordination, the cost optimization strategy of dual-channel began to be more inclined to the trade cost of cross-border enterprises, and its main focus was on transportation costs and tariff barriers. For all enterprises involved in the cross-border supply chain, cost optimization is an important goal for the sustainable operation and development of enterprises, so we need to pay attention to how to further improve the sustainable operation of the supply chain through optimization decisions.

In fact, the development strategy and decision-making of cross-border dual-channel supply chain are more complex than that of non-cross-border supply chain under the same conditions, not only in the competition between manufacturers and retailers, but also in the consideration of market demand and channel competition, and the decision-making scope needs to be considered more broadly. Based on this condition, the manufacturer should constantly adjust the strategy in the cross-border supply chain to complete the optimal decision of revenue. In general, the problems in dual-channel supply chain are mostly explored by using relevant game theory models, and many deductions based on the pricing game between manufacturers and retailers are put forward [10], so there are sufficient theoretical references in the part of channel competition and channel coordination. However, the current research on relevant policies and strategic pricing shows a certain lack. This paper considers the dual-channel supply chain, and takes tariff, export tax refund and transportation heterogeneity conditions as the key factors and decision-making environment, in an attempt to provide an effective theoretical reference in this field.

In summary, this paper aims to find answers to the following questions:

1. Considering the basic background of cross-border dual-channel supply chain, which decision-making mode is more conducive to the profit acquisition of manufacturers and retailers?
2. In the dual-channel model, what pricing strategies can manufacturers and retailers adopt in the face of tariff, export tax rebate and transportation heterogeneity to achieve a win-win situation?
3. What impact do macro-policy conditions (tariffs, export tax rebates) have on supply chain decision-making?

In order to solve the above questions, this paper introduces transport heterogeneity, trade tariffs and export tax rebates into the cross-border dual-channel supply chain model at the same time, and establishes a two-stage Stackelberg game decentralized decision-making model with manufacturers and retailers as the main body and a centralized decision-making model with supply chains as the main body. This model can solve and analyze the influence of tariff, transportation heterogeneity and export tax refund on the optimal pricing analysis of this supply chain and the optimal evaluation criteria of demand under the two situations. In addition, this paper also analyzes the influence of the profits and benefits of the internal members of the whole supply chain and discusses the results according to the analysis.

This paper intends to arrange the writing according to the following process. Section 2 reviews the existing literature basis in related research fields; Section 3 proposes hypothesis and model construction; Section 4 solves the model. In Section 5, the model parameters are simulated. Section 6 describes management insights and practical implications, as well as its potential limitations and future prospects.

## 2. Literature review

### 2.1 Transportation costs, e-commerce and transnational supply chains

Transnational supply chain has been extensively researched by domestic and foreign scholars [11]. Take into account the transport process and variable transport costs, Zhang studied the global supply chain and proposed an optimization model for global supply chain management, considering the uncertainties of demand, transportation cost allocation, tax rate, etc [12]. At the same time, how to manage the sustainable supply chain has also become A challenge. By constructing a general three-level supply chain model, the decision optimization is carried out in the case of direct and indirect transportation [13]. Gustav Albertzeth conducted a strategic assessment of traffic disruption in the context of transport and summarized four mitigation strategies: risk acceptance, redundant inventory, flexible routing and redundancy—flexibility [14]. Santanu Kumar Ghosh conducted an analysis and research on the shift of consumers and sellers to digital e-commerce after the coronavirus pandemic (COVID-19). Through modeling a two-level supply chain under random demand, he found that under decentralized decision-making, universal power can affect the selling price of products. Make it increase, and at the same time reduce the total profit of the supply chain [15].

Xiaoli Yan This paper discusses the construction and channel of transnational supply chain, and constructs a robust model of profit maximization motivation based on different channel scenarios at home and abroad [16]. Ogunranti analyzes a decentralized global supply chain under a newsvendor setting, where a supplier delivers a certain quantity of a single product to a buyer in accordance with the terms of a mutually agreed upon contract. This contract is signed prior to the delivery of the product and subsequent payment, thus, exposing the supply chain to the risk of currency exchange rate fluctuations [17]. Hsu and Zhu designed Chinese export-oriented tax and tariff rules to study their impact on the optimal supply chain design and operation of companies that produce products in China and sell them in China and abroad [18].

Using entropy weight method (EWM), simple additive weight method (SAW) and interpretive structure model (ISM), Dewan Hafiz Nabil integrated the methods to build a model that can detect the supply chain elasticity of transnational e-commerce, and believed that supply chain elasticity has a growth effect on the development of e-commerce enterprises under transnational background [19]. Deli Wang built a dual-channel supply chain composed of cross-border suppliers, cross-border e-commerce enterprises, retailers and consumers on the basis of consumer satisfaction, and studied the benefit and cost sharing contract of supply chain under centralized and decentralized decision-making conditions [20]. Hao Zhang proved the effectiveness of the cross-border e-commerce supply chain balance assessment model through empirical research, and believed that cross-border e-commerce (CBEC) enterprises should maintain a proper balance between supply chain elasticity and vulnerability [21]. Dewan Hafiz Nabil believes that capital and taxation are the most important factors in the financing and operation of transnational supply chains, and discusses supply chain strategy selection and contract clause setting through Stackelberg game method. In the context of the development of economic globalization, risk diffusion of transnational supply chains is particularly important [19]. Zhimei Lei found that individual differences and network structure are easy to be ignored in the study of supply chain risk transmission, and there are limitations. Based on this, he proposed an improved susceptibility—infection-susceptibility (SIS) model. Four characteristics of risk transmission in transnational supply chain are pointed out in optimization and analysis [22].

### 2.2 The impact of tariff policy on transnational supply chain

The impact of tariffs on transnational supply chains is complex and multifaceted. The increase of tariff will increase the cost, increase the market access barriers and increase the uncertainty, which will affect the competitiveness of enterprises and the business environment. The research on tariff also has different directions, starting from the negative impact at the beginning, but with the deepening of the research, more positive studies have been highlighted. Rong studied the impact of manufacturers’ altruism preference and government subsidies on transnational green supply chain under dynamic tariff, and the results showed that with the increase of tariff rate, altruism preference or government subsidies could promote the development of green supply chain [18]. Ludema found that the product quality and export price of American companies would improve with the reduction of overseas tariffs [23]. Fan Based on the data of Chinese enterprises, the study concluded that considering productivity, reducing input tariffs would prompt existing importers and exporters to raise their product prices [24].

The importance of tariff policies continues to be reflected in transnational supply chains, and tariff fluctuations to a large extent affect the stability of supply chain decisions. In this context, Xiaoyang Zhou discussed the joint impact of carbon tariffs in different carbon control countries, and found that manufacturers with low carbon emission reduction costs would further increase their product output and profits with the increase of carbon tariffs. And put forward the win-win strategy in the case of emission reduction cost difference [25]. Su Yi established Stackelberg game model to discuss transnational green supply chain decision-making under two situations of government subsidy background [26]. Omar Alhawari examines the impact of tariffs on the manufacturing supply chain under consideration, starting with the risk of government tariffs on imported goods [27].

Under different models, both manufacturers and retailers need to consider the impact of tariffs. Wei Li established three remanufacturing models for original equipment manufacturers (Oems) in exporting countries, analyzed their optimal pricing and carbon emission reduction decisions, and studied the impact of different levels of carbon tax and tariff combinations on decision-making and the environment [28]. Economic policy determines many tariff barriers, Xiao Hu has studied the combined supply chain of overseas manufacturers, domestic suppliers and third-party international logistics service providers, pointing out that it is difficult to achieve coordination of supply chains when transportation costs and insurance costs are taken into account in tariff costs [29]. E-commerce platforms promote the information flow of cross-border supply chains, in which tariffs on cross-border commodity flows become the subject of supply chain impacts, mainly affecting supply chain network, distribution channel structure, product quantity and quality, production outsourcing, procurement patterns and supply chain emissions. In addition to decision-making, there is also a certain reference to the dynamic changes between relevant policies and transnational supply chains. Under the background of localization system, American manufacturers are more inclined to reduce the spatial complexity of the supply base, and they are more dependent on foreign suppliers and less dependent on local suppliers, showing a trend contrary to the government policy objectives [30].

Regarding export tax rebate, Braakmann took China as an example to study how the adjustment of export tax rebate rate affects export prices [31]. Bai considered the supply chain decision of government subsidise and VAT refunds, and concluded that VAT refund would positively affect manufacturers [32]. Chen believe that the export tax rebate policy mainly promotes the growth effect of China’s exports [33].

As one of the most important trade policies, export tax rebate plays an important role in the development of enterprise industry and supply chain. Yue Liang used the sample data from 2001 to 2013 to verify the relationship between tax rebate, enterprise innovation and product quality in China’s agricultural product processing industry, and found that there was a transmission effect. By increasing the tax rebate rate, it can promote the innovation and development of enterprises and the improvement of product quality to a certain extent [34]. On the other hand, Fuchang Li pointed out that the export tax rebate policy can mitigate the negative impact of import tariffs, thus promoting manufacturers and retailers to maximize the optimal decision [35]. Bochun Shao believes that China’s export tax rebate policy has a positive role in promoting export growth, and its impact is mainly reflected in the export volume and the structure of export commodities [36].

### 2.3 Dual channel supply chain

Cross-border dual-channel supply chains have been a concern for a long time. Ding studied the dual-channel problem between a manufacturer and a retailer. Taking the manufacturer as the leader of Stackelberg, the research results showed that dual-channel operation was optimal for the manufacturer only under certain conditions [37]. Manufacturers dominate the Stackelberg game, according to Cattani [38]. Sharma established a manufacturer-led Stackelberg game model in which retailers’ fairness concerns influenced supply chain decisions [39]. Chakraborty used the Stackelberg game model for two different situations of manufacturer dominance and retailer dominance, established a Nash game model of equal rights for both manufacturers and retailers, and compared and analyzed the results of the two game models with data [40]. Hu studied dual-channel supply chain decision-making under centralized decision making and decentralized decision making respectively. According to the research, centralized decision making will obtain more total profits of the supply chain within a certain range of revenue sharing ratio [41]. Wong studied the dual-channel supply chain decision making under the circumstances of decentralized decision making and centralized decision making based on uniform pricing, and found that compared with decentralized decision making, centralized decision making has more obvious advantages, mainly reflected in the obvious increase in the total profit of the supply chain [42].

At present, the academia has a relatively complete study on the price, channel, mechanism and decision-making of supply chain under different conditions. Dual-channel is favored by academia and manufacturers in this environment. Santanu Kumar Ghosh believes that retailers and manufacturers can have a wider choice in the range of dual channels, depending on customer preference [43]. Subhendu Ruidas examines different inventory pricing decisions in the context of demand disruption and price correction, as well as the optimal inventory policy under cap-and-trade regulation [44]. Based on the environmental context, Santanu Kumar Ghosh also proposed a two-level two-channel supply chain model under emission sensitive demand [45]. When reviewing the previous literature, it can be seen that most scholars seldom consider the complexity of transnational supply chains, and only study from unilateral and few aspects [46–49]. Few literatures comprehensively analyze the impact of transportation heterogeneity, trade tariffs and export tax rebates on cross-border dual-channel supply chain decision-making. Table 1 shows the comparison between the model proposed in the existing literature and the model proposed in this paper.

**Table 1:** The model proposed in this paper is compared with the existing models in the relevant literature.

Author	Channel used	Cross-border e-commerce model	Scenarios	Tariff policy and transportation costs	Construction subject
Fuchang Li [35]	Dual channel	Yes	Centralized and decentralized	Consider tariff policy separately	Manufacturers and retailers
Su Yi [26]	Retail channel	No	Centralized decision	Consider tariff policy separately	Manufacturers and retailers
Santanu Kumar Ghosh [15]	Dual channel	No	Centralized and decentralized	No	consumer
Deli Wang [20]	Dual channel	Yes	Centralized and decentralized	No	Suppliers, e-commerce companies, retailers and consumers
Sharma [39]	Dual channel	Yes	Centralized and decentralized	No	Manufacturers and retailers
Hu [41]	Retail channel	No	Centralized decision	No	Suppliers and consumers
Use in this paper	Dual channel	Yes	Centralized and decentralized	All include	Manufacturers, retailers and consumers

### 3. Hypothesis and model design

Based on the condition of differential pricing, build a two-level cross-border e-commerce dual-channel supply chain system:

There are three main bodies in this model, which are domestic manufacturers, foreign retailers, and finally foreign consumers [50]. The domestic manufacturer manufactures the products in China at a unit cost of c. The manufacturer provides the products to overseas consumers through the cross-border e-commerce platform at the direct price *p*_*m*_ [51]. In addition, manufacturers wholesale goods at the wholesale price w, and retailers sell them to overseas consumers at the retail price *p*_*r*_, where *p*_*m*_ ≠ *p*_*r*_.

**Hypothesis 1:** The domestic manufacturer needs to bear the international transportation cost of *s*_1_, per unit of product sold directly to overseas consumers through cross-border e-commerce; The international transportation cost per unit of product for overseas retailers to import products from domestic manufacturers is *s*_2_. Meanwhile, since the domestic transportation cost of retailers is very small compared with the international transportation cost [52], the domestic transportation cost of retailers is not considered in this paper, where *s*_1_ > *s*_2_.
**Hypothesis 2:** When an overseas retailer imports a unit product, it is subject to tariff t, while the retailer usually transfers the tariff cost to the consumer through the tax-included price *p*_*r*_ [35], that is, the final retail price of the product includes tariff t, without considering other costs and taxes.
**Hypothesis 3:** For the convenience of calculation, foreign consumers and foreign retailers need to pay the same tariff for importing a unit product, while domestic manufacturers often transfer the tariff cost to foreign consumers through the tax-included price *p*_*m*_ [35], that is, foreign consumers do not need to pay the tariff separately.
**Hypothesis 4:** According to the operating principle and function of China’s export tariff [53], it is assumed that the export goods of the manufacturer will be refunded according to the export tax rebate rate based on the cost of the export goods [35], and *d* is the VAT rebate rate implemented by the government on the export link of the manufacturer.

Due to the uncertainty of the market, there are random market demands in the two channels at the same time, which jointly reflects the randomness of the market demand for the two channels. The specific values are set as shown in the following (Table 2):

**Table 2:** Main variables.

Variable	Definition
*c*	Unit Cost
*p*_*m*_	Direct Selling Price
*w*	Wholesale Price
*p*_*r*_	Retail Price (Inclusive of Tax)
*s*_1_	International Shipping Cost per Unit for Domestic Manufacturers Selling Abroad via Cross-border E-commerce
*s*_2_	International Shipping Cost per Unit for Foreign Retailers Importing from Domestic Manufacturers
*t*	Tariff
*d*	VAT Rebate Rate for Manufacturers on Exports
*D*_*m*_	Demand for Manufacturer’s Cross-border E-commerce Direct Sales Channel
*D*_*r*_	Demand for Retailer’s Traditional Retail Channel
ρ	Consumer Preference Coefficient for Cross-border E-commerce Direct Sales Channel (ρ≥0)
(1 − *ρ*)	Consumer Preference Coefficient for Traditional Retail Channel
*a*	Total Market Demand for Overseas Direct Selling and Retail Channels
*b*	Consumer Price Sensitivity Coefficient (b>0)
*γ*	Impact Rate of Brick-and-Mortar and Online Platform Prices on Each Other’s Product Demand (b>γ>0)
*ε*_*m*_	Uncertain Demand
*ε*_*r*_	Certain Demand

Then the expected demand function *D*_*m*_ and *D*_*r*_ of the manufacturer and retailer are respectively:

$$D_{m}=\rho \alpha -bp_{m}+\gamma p_{r}+\mu_{m}$$ (1)

$$D_{r}=\left(1-\rho \right)\alpha -bp_{r}+\gamma p_{m}+\mu_{r}$$ (2)

Then the decision objective function of the manufacturer and the retailer is:

$$\pi_{m}=\left[p_{m}-s_{1}-t-\left(1-d\right)c\right]D_{m}+\left[w-\left(1-d\right)c\right]D_{r}$$ (3)

$$\pi_{r}=p_{r}D_{r}-wD_{r}-s_{2}D_{r}-tD_{r}$$ (4)

## 4. Model solving

### 4.1 Decentralized decision making

The participants in the supply chain include a domestic manufacturer and an overseas retailer, and they play the Stackelberg game. In the first stage, domestic manufacturers take maximizing their own profits as the goal to decide the product wholesale price ***W*** and cross-border e-commerce direct sales channel sales price *p*_*m*_; In the second stage, zero overseas sellers decide the sales price of traditional retail channels *p*_*r*_ with the goal of maximizing their own profits.

Proposition 1: When *b* > *γ*, the optimal decision for manufacturers and retailers is:

$${\text{w}}^{*}=\frac{ab+b\mu_{r}+\gamma \mu_{m}+\left(b^{2}-\gamma^{2}\right)\left(c-s_{2}-t-dc\right)-ab\rho +a\gamma \rho }{2b^{2}-2\gamma^{2}}$$ (5)

$${p_{m}}^{*}=\frac{a\gamma +\gamma \mu_{r}+b\mu_{m}+\left(b^{2}-\gamma^{2}\right)\left(c+s_{1}+t-dc\right)+ab\rho -a\gamma \rho }{2b^{2}-2\gamma^{2}}$$ (6)

$$\begin{array}{c} {p_{r}}^{*}=\frac{b\left(b^{2}-\gamma^{2}\right)\left(c+s_{2}+t+dc\right)+3b^{2}\mu_{r}+\gamma \left(b^{2}-\gamma^{2}\right)\left(c+s_{1}+t+dc\right)-\gamma^{2}\mu_{r}}{4b\left(b^{2}-\gamma^{2}\right)}\\ +\frac{\left(3ab^{2}-a\gamma^{2}\right)\left(1-\rho \right)+2b\gamma \mu_{m}+2ab\gamma \rho }{4b\left(b^{2}-\gamma^{2}\right)} \end{array}$$ (7)

**Proof:** Using backward induction method to solve, first according to Eq (4) to find the retailer’s first derivative: $\frac{\partial \pi_{r}}{\partial p_{r}}=-bp_{\text{r}}^{2}+\left[\left(1-\rho \right)\alpha +\gamma p_{m}+\mu_{\text{r}}\right]p_{r}+\left(t+\text{w}+s_{2}\right)bp_{r}$. Since $\frac{\partial^{2}\pi_{r}}{\partial {p_{r}}^{2}}=-2b<0$, *π*_*r*_ is a concave function of *p*_*r*_. Let (∂π_r)/(∂p_r) = 0, can get $p_{r}=\frac{\left(1-\rho \right)\alpha +\gamma p_{m}+\gamma t+\mu_{r}}{2b}+\frac{t+w+s_{2}}{2}$. Taking *p*_*r*_ into Eq (3) and then get *w*, *p*_*m*_ first derivative $\frac{\partial \pi_{m}}{\partial w},\frac{\partial \pi_{m}}{\partial p_{m}}$ respectively. Then the second derivative of *w and p*_*m*_ can get Hesse matrix $H=\left[\begin{array}{cc} \frac{\gamma^{2}-2b^{2}}{b} & \gamma \\ \gamma & -b \end{array}\right]$. Therefore, when 2*b*^2^ − 2*γ*^2^ > 0, that is, *b* > *γ*, the Hessian matrix ***H*** is negative. That is, the manufacturer’s profit *π*_*m*_ is a concave function of *w and p*_*m*_. $\frac{\partial \pi_{m}}{\partial w},\frac{\partial \pi_{m}}{\partial p_{m}}$ is equal to zero, to solve the available, w*, *p*_m_*, then, w*, *p*_m_* in *p*_*r*_, the reduction can be *p*_r_*. Proposition 1 is proved.

From proposition 1, it is further obtained that the optimal market demand of traditional retail channels, the optimal market demand of cross-border e-commerce direct sales channels, the optimal profit of manufacturers and the optimal profit of retailers are as follows:

$${D_{m}}^{*}=\frac{a\gamma +\gamma \mu_{r}+2b\mu_{m}+b\gamma \left(c+s_{2}+t-dc\right)+\left(\gamma^{2}-2b^{2}\right)\left(c+s_{1}+t-dc\right)+2ab\rho -a\gamma \rho }{4b}$$ (8)

$${D_{r}}^{*}=\frac{a+\mu_{r}-bc-a\rho -bs_{2}-bt+c\gamma +\gamma s_{1}+t\gamma +bdc+\gamma dc}{4}$$ (9)

$$\begin{array}{ll} {\pi_{m}}^{*} & =\frac{\left(a+\mu_{r}-bc-a\rho -bs_{2}-bt+c\gamma +\gamma s_{1}+t\gamma +bdc+\gamma dc\right)}{4}\\ & *\frac{\left[ab+b\mu_{r}+\gamma \mu_{m}+\left(-b^{2}+\gamma^{2}\right)\left(c+s_{2}+t-dc\right)-ab\rho +a\gamma \rho \right]}{2b^{2}-2\gamma^{2}}\\ & +\frac{ab+b\mu_{r}+\gamma \mu_{m}+\left(b^{2}-\gamma^{2}\right)\left(c-s_{2}-t-dc\right)-ab\rho +a\gamma \rho }{2b^{2}-2\gamma^{2}}\\ & *\frac{\left[a\gamma +\gamma \mu_{r}+2b\mu_{m}+b\gamma \left(c+s_{2}+t-dc\right)+\left(\gamma^{2}-2b^{2}\right)\left(c+s_{1}+t-dc\right)+2ab\rho -a\gamma \rho \right]}{4b} \end{array}$$ (10)

$${\pi_{r}}^{*}=\frac{{\left[\alpha \left(1-\rho \right)+\mu_{r}-b\left(c+s_{2}+t-dc\right)+\gamma \left(c+s_{1}+t-dc\right)\right]}^{2}}{16b}$$ (11)

**Remark 1:** In cross-border dual-channel supply chains, there are: 1) $\frac{\partial {p_{m}}^{*}}{\partial t}>0$, $\frac{\partial w^{*}}{\partial t}<0$, $\frac{\partial {p_{r}}^{*}}{\partial t}>0$; When $b\geq \gamma ,\left|\frac{\partial {p_{m}}^{*}}{\partial t}\right|=\left|\frac{\partial w^{*}}{\partial t}\right|\geq \left|\frac{\partial {p_{r}}^{*}}{\partial t}\right|$. 2) $\frac{\partial {D_{m}}^{*}}{\partial t}<0$, $\frac{\partial {D_{r}}^{*}}{\partial t}<0$, $\left|\frac{\partial {D_{m}}^{*}}{\partial t}\right|-\left|\frac{\partial {D_{r}}^{*}}{\partial t}\right|\geq 0$.

Remark 1 shows that: 1) With the increase of tariff, the wholesale price of domestic manufacturers will decrease, while the direct selling price of manufacturers and the retail price of foreign retailers will both increase, indicating that tariff changes will affect the price setting of the supply chain. However, the impact of tariff changes on the price decisions of manufacturers and retailers is inconsistent and is related to consumer price sensitivity coefficient and demand elasticity among channels [54]. When the sensitivity coefficient of consumer price is higher than the elasticity of demand between channels, tariff changes have a bigger effect on manufacturers’ price decisions than on retailers. This means that tariffs are currently making it harder for manufacturers to set prices; 2) With the increase of tariffs, the demand for cross-border e-commerce channels and the demand for traditional retail channels will decrease, which indicates that the tariff changes are not conducive to import and export. Moreover, the impact of tariffs on the demand of direct sales channels is greater than that of retail channels, which means that tariff changes have a greater impact on the demand of cross-border e-commerce channels [55]. Compared with overseas retailers, domestic manufacturers will bear a greater blow.

**Remark 2:** double channels in cross-border supply chain, there are: 1) when 0 < t < *t*_1_, $\frac{\partial {\pi_{m}}^{*}}{\partial \text{t}}<0$, when $\text{t}>t_{1},\frac{\partial {\pi_{m}}^{*}}{\partial \text{t}}>0$; When 0 < t < *t*_2_, $\frac{\partial {\pi_{r}}^{*}}{\partial \text{t}}<0$, when t > *t*_2_时, $\frac{\partial {\pi_{m}}^{*}}{\partial \text{t}}>0$; 2) $\frac{\partial^{2}{\pi_{m}}^{*}}{\partial {\text{t}}^{2}}>0$, $\frac{\partial^{2}{\pi_{r}}^{*}}{\partial {\text{t}}^{2}}>0$.


$$\begin{array}{c} t_{1}=\frac{\left(2b^{2}-\gamma^{2}\right)\left(cd-c-s_{1}\right)+b^{2}\left(cd-c-s_{2}\right)+2b\gamma \left(c-cd\right)+2b\mu_{m}}{3b^{2}-2b\gamma -\gamma^{2}}\\ +\frac{b\left(a+a\rho +\mu_{r}\right)+\gamma \left(a-a\rho +\mu_{r}\right)+b\gamma \left(s_{1}+s_{2}\right)}{3b^{2}-2b\gamma -\gamma^{2}} \end{array}$$

$$t_{2}=\frac{b\left(cd-c-s_{2}\right)-\gamma \left(cd-c-s_{1}\right)+a-a\rho +\mu_{r}}{b-\gamma }$$

Remark 2 shows that when the tariff is lower than the threshold t_1, the manufacturer’s profit will generally decrease with the increase of the tariff, but when the tariff is higher than the threshold t_1, the further increase of the tariff may lead to the increase of the manufacturer’s profit. However, combined with inference 1, it can be seen that the dual-channel demand at this time is already negative, so the profit must be negative, and there is no reference significance. When the tariff is lower than the threshold t_2, the retailer’s profit will decrease with the increase of the tariff; Similarly, when the tariff is higher than the threshold t_2, it has no reference significance. In summary, as tariffs increase, manufacturers’ profits and retailers’ profits will decrease, which means that tariffs will squeeze manufacturers’ and retailers’ profits, which is not conducive to imports and exports.

**Remark 3:** In cross-border dual-channel supply chains, there are: $\frac{\partial {\text{w}}^{*}}{\partial \text{d}}<0$, $\frac{\partial {p_{m}}^{*}}{\partial \text{d}}<0$, $\frac{\partial {p_{r}}^{*}}{\partial \text{d}}<0$; $\left|\frac{\partial {p_{m}}^{*}}{\partial d}\right|=\left|\frac{\partial {p_{w}}^{*}}{\partial d}\right|\geq \left|\frac{\partial {p_{r}}^{*}}{\partial d}\right|$ 2) $\frac{\partial {D_{m}}^{*}}{\partial d}>0$, $\frac{\partial {D_{r}}^{*}}{\partial d}>0$, $\left|\frac{\partial {D_{m}}^{*}}{\partial d}\right|-\left|\frac{\partial {D_{r}}^{*}}{\partial d}\right|\geq 0$.

Remark 3 shows that, 1) With the increase of export tax rebate rate, the direct selling price, wholesale price and retail price of overseas retailers will decrease, which indicates that the change of export tax rebate will affect the pricing of the supply chain. It is worth noting that the impact of export tax rebate changes on direct selling prices, wholesale prices and retail prices is inconsistent, and the impact of export tax rebate changes on manufacturers’ pricing decisions is greater than that of retailers, indicating that the existence of export tax rebate is more favorable to manufacturers’ pricing. 2) With the increase of export tax rebate rate, the demand of cross-border e-commerce channels and traditional retail channels will increase, which indicates that export tax rebate is conducive to import and export. Moreover, the impact of export tax rebate on the demand of direct sales channels is greater than that of retail channels, which means that export tax rebate has a greater impact on the demand of domestic manufacturers. Compared with overseas retailers, domestic manufacturers will greatly increase the export volume.

**Remark 4:** In cross-border dual-channel supply chains, there is: 1) when *d* < *d*_1_ < 0, $\frac{\partial {\pi_{m}}^{*}}{\partial \text{d}}<0$, when d > 0, $\frac{\partial {\pi_{m}}^{*}}{\partial \text{d}}>0$; when d < *d*_2_ < 0, $\frac{\partial {\pi_{r}}^{*}}{\partial \text{d}}<0$, when d > *d*_2_, $\frac{\partial {\pi_{m}}^{*}}{\partial \text{d}}>0$; 2) $\frac{\partial^{2}{\pi_{m}}^{*}}{\partial {\text{d}}^{2}}>0$, $\frac{\partial^{2}{\pi_{r}}^{*}}{\partial {\text{d}}^{2}}>0$.

Remark 4 shows that when the export tax rebate rate is lower than the threshold value d_1, the manufacturer’s profits will decrease with the increase of the export tax rebate rate, but this is not in line with the reality and has no reference significance. When the export tax rebate rate is greater than 0, the manufacturer’s profit will increase with the increase of the export tax rebate rate. Similarly, when the export tax rebate rate is less than 0, it has no reference significance; When the export tax rebate rate is greater than 0, the export tax rebate rate is positively correlated with the retailer’s profit and income, and the value changes gradually increase. In short, the profits of manufacturers and retailers are affected by the increase of export tax rebates, and both are increasing functions.

**Remark 5**: In cross-border dual-channel supply chains, there is: 1) $\frac{\partial {p_{m}}^{*}}{\partial s_{1}}>0$, $\frac{\partial {p_{r}}^{*}}{\partial s_{1}}>0$, $\frac{\partial {\text{w}}^{*}}{\partial s_{1}}=0$, $\frac{\partial {p_{m}}^{*}}{\partial s_{2}}=0$, $\frac{\partial {p_{r}}^{*}}{\partial s_{2}}>0$, $\frac{\partial {\text{w}}^{*}}{\partial s_{2}}<0$;

Remark 5 shows that as the unit freight of the direct selling channel increases, the direct selling price and retail price will increase, while the wholesale price will not change. As the retail channel unit freight increases, the wholesale price will decrease, the retail price will increase, and the direct sales price will not change. It is worth noting that the change of unit freight rate of direct channel will affect the pricing decision of overseas retailers and is positive, and the change of unit freight rate of retail channel will also affect the pricing of manufacturers, but only affect the wholesale price and is negative.

**Remark 6:** In cross-border dual-channel supply chains, there is: 1) $\frac{\partial {D_{m}}^{*}}{\partial s_{1}}<0$, $\frac{\partial {D_{r}}^{*}}{\partial s_{1}}>0$, $\frac{\partial {D_{m}}^{*}}{\partial s_{2}}>0$, $\frac{\partial {{\text{D}}_{r}}^{*}}{\partial s_{2}}<0$; $\left|\frac{\partial {D_{m}}^{*}}{\partial s_{1}}\right|\geq \left|\frac{\partial {{\text{D}}_{r}}^{*}}{\partial s_{2}}\right|\geq \left|\frac{\partial {D_{r}}^{*}}{\partial s_{1}}\right|=\left|\frac{\partial {D_{m}}^{*}}{\partial s_{2}}\right|$ 2) $\frac{\partial {\pi_{m}}^{*}}{\partial s_{1}}<0$, $\frac{\partial {\pi_{r}}^{*}}{\partial s_{1}}>0$, $\frac{\partial {\pi_{m}}^{*}}{\partial s_{2}}<0$, $\frac{\partial \pi^{*}}{\partial s_{2}}<0$.

Remark 6 shows that with the increase of unit freight of direct selling channel, the demand of direct selling channel will decrease while the demand of retail channel will increase. With the increase of unit freight of retail channels, the demand for direct sales channels will increase, and the demand for retail channels will decrease. The impact of unit freight changes on the demand of manufacturers and retailers is inconsistent, and is related to the consumer price sensitivity coefficient and the demand elasticity among channels. Combined with inference 2 and inference 4, we discard the result that has no reference significance, then with the increase of unit freight of direct selling channel, the profit of direct selling channel will decrease, while the profit of retail channel will increase. With the increase of unit freight of retail channels, the profits of direct sales channels will decrease, and the profits of retail channels will decrease. Explain how changes in unit freight rates affect the profits of manufacturers and retailers.

### 4.2 Centralized decision model

This part studies whether centralized decision making can optimize supply chain decision making. In this model, manufacturers and retailers jointly pursue the maximum relevant profits of the entire dual-channel supply chain, and set *π*^*C*^ to act as the expression of the profit function of the entire dual-channel supply chain under the centralized decision mode, and split the profit function of manufacturers and retailers under the centralized decision into $\pi_{m}^{C}$ and $\pi_{r}^{C}$. The superscript C stands for centralized decision making. Hence:

$$D_{m}^{C}=\rho \alpha -bp_{m}^{C}+\gamma p_{r}^{C}+\mu_{m}$$ (12)

$$\begin{array}{c} D_{r}^{C}=\left(1-\rho \right)\alpha -bp_{r}^{C}+\gamma p_{m}^{C}+\mu_{r}\\ \pi^{C}=\left[p_{m}^{C}-s_{1}-t-\left(1-d\right)c\right]D_{m}^{C}+\left[p_{r}^{C}-t-s_{2}-\left(1-d\right)c\right]D_{r}^{C} \end{array}$$ (13)

**Proposition 2**: Centralized decision model, the optimal decision for manufacturers and retailers is:

$${p_{m}^{C}}^{*}=\frac{a\gamma +\gamma \mu_{r}+b\mu_{m}+\left(b^{2}-\gamma^{2}\right)\left(c+s_{1}+t-dc\right)+ab\rho -a\gamma \rho }{2b^{2}-2\gamma^{2}}$$ (14)

$${p_{r}^{C}}^{*}=\frac{ab+b\mu_{r}+\gamma \mu_{m}+\left(b^{2}-\gamma^{2}\right)\left(c-s_{2}-t-dc\right)-ab\rho +a\gamma \rho }{2b^{2}-2\gamma^{2}}$$ (15)

**Prove:** based on *π*^*C*^ respectively about $p_{m}^{C},p_{r}^{C}$ the first derivative of derivation concluded $\frac{\partial \pi^{C}}{\partial p_{m}^{C}}=0,\frac{\partial \pi^{C}}{\partial p_{r}^{C}}=0$. Then, the second derivative of $p_{m}^{C},p_{r}^{C}$ is obtained, and the Hessian matrix ${\text{H}}^{C}=\left[\begin{array}{cc} -2b & 2\gamma \\ 2\gamma & -2b \end{array}\right]$ is obtained. Therefore, when 4*b*^2^ − 4*γ*^2^ > 0, that is, when *b* > *γ*, the Hessian matrix H^*C*^ is negative definite. That is, the profit *π*^*C*^ of the supply chain is a concave function of *p*_*m*_, *p*_*r*_. $\frac{\partial \pi^{C}}{\partial p_{m}^{C}},\frac{\partial \pi^{C}}{\partial p_{r}^{C}}$ equal to zero, to solve the available ${p_{m}^{C}}^{*},{p_{r}^{C}}^{*}$, the proposition 2 is proved.

From proposition 2, it is further obtained that the optimal market demand of traditional retail channels, the optimal market demand of cross-border e-commerce network direct sales channels, and the total profit of supply chain are respectively.


$${D_{m}}^{*}=\frac{\mu_{m}-bc+a\rho -bs_{1}-bt+c\gamma +\gamma s_{2}+t\gamma +bdc-\gamma dc}{2}$$ (16)

$${D_{r}}^{*}=\frac{a+\mu_{r}-bc-a\rho -bs_{2}-bt+c\gamma +\gamma s_{1}+t\gamma +bdc-\gamma dc}{2}$$ (17)

$$\begin{array}{ll} {\pi^{C}}^{*} & =\frac{\mu_{m}-bc+a\rho -bs_{1}-bt+c\gamma +\gamma s_{2}+t\gamma +bdc-\gamma dc}{2}\\ & *\frac{a\gamma +\gamma \mu_{r}+b\mu_{m}+\left(b^{2}-\gamma^{2}\right)\left(c+s_{1}+t-dc\right)+ab\rho -a\gamma \rho }{2b^{2}-2\gamma^{2}}\\ & +\frac{ab+b\mu_{r}+\gamma \mu_{m}+\left(b^{2}-\gamma^{2}\right)\left(c-s_{2}-t-dc\right)-ab\rho +a\gamma \rho }{2b^{2}-2\gamma^{2}}\\ & *\frac{a+\mu_{r}-bc-a\rho -bs_{2}-bt+c\gamma +\gamma s_{1}+t\gamma +bdc-\gamma dc}{2} \end{array}$$ (18)

**Remark 7**: Centralized decision making exists as opposed to decentralized decision making, there is: 1) $\frac{\partial {p_{m}^{C}}^{*}}{\partial t}>0$, $\frac{\partial {p_{r}^{C}}^{*}}{\partial t}>0$, $\left|\frac{\partial {p_{m}^{C}}^{*}}{\partial t}\right|=\left|\frac{\partial {p_{r}^{C}}^{*}}{\partial t}\right|$, $\frac{\partial {D_{m}^{C}}^{*}}{\partial t}<0$, $\frac{\partial {D_{r}^{C}}^{*}}{\partial t}<0$, $\left|\frac{\partial {D_{m}^{C}}^{*}}{\partial t}\right|=\left|\frac{\partial {D_{r}^{C}}^{*}}{\partial t}\right|$; 2) $\frac{\partial {p_{m}^{C}}^{*}}{\partial d}<0$, $\frac{\partial {p_{r}^{C}}^{*}}{\partial d}<0$; $\left|\frac{\partial {p_{m}^{C}}^{*}}{\partial d}\right|=\left|\frac{\partial {p_{r}^{C}}^{*}}{\partial d}\right|$, $\frac{\partial {D_{m}^{C}}^{*}}{\partial d}>0$, $\frac{\partial {D_{r}^{C}}^{*}}{\partial d}>0$, $\left|\frac{\partial {D_{m}^{C}}^{*}}{\partial d}\right|=\left|\frac{\partial {D_{r}^{C}}^{*}}{\partial d}\right|$.

Remark 7 shows that the centralized decision-making model and the decentralized decision-making model have in common that tariffs are positively correlated to the changes of the two kinds of prices, tariffs have opposite changes in the demand for direct selling channels and retail channels, export tax rebates have negative changes in the direction of direct selling prices and retail prices, and tariffs have positive changes in the demand for direct selling channels and retail channels. The difference is that under centralized decision making, the influence of tariff and export tax rebate on all optimal decisions of manufacturers and retailers is the same, which indicates that centralized decision making has an optimization effect on supply chain decision making.

**Remark 8:** the related advantage of centralized decision making for decentralize decision making is that ${{\text{p}}_{\text{m}}^{\text{C}}}^{*}-{{\text{p}}_{\text{m}}}^{*}=0,{{\text{p}}_{\text{r}}^{\text{C}}}^{*}-{{\text{p}}_{\text{r}}}^{*}=\frac{-\left[\alpha +\mu_{\text{r}}-\text{b}\left(\text{c}+{\text{s}}_{2}+\text{t}-\text{dc}\right)+\gamma \left(\text{c}+{\text{s}}_{1}+\text{t}-\text{dc}\right)\right]}{4\text{b}}<0$.

Remark 8 shows that the difference between the centralized decision-making model and the decentralized decision making model is that the direct selling price does not change, but under the centralized decision making, the retailer’s price will decrease. This shows that under decentralized decision-making, retailer prices may be high, while centralized decision-making can better optimize the optimal pricing decision of the supply chain.

**Remark 9: ${D_{m}^{C}}^{*}-{D_{m}}^{*}<0$**, ${D_{r}^{C}}^{*}-{D_{r}}^{*}=\frac{-\left[\alpha +\mu_{r}-b\left(c+s_{2}+t-dc\right)+\gamma \left(c+s_{1}+t-dc\right)\right]}{4b}>0$, ${D_{m}^{C}}^{*}+{D_{r}^{C}}^{*}>{D_{m}}^{*}+{D_{r}}^{*}$; $\pi^{C}-\pi =\frac{{\left[\alpha \left(1-p\right)+\mu_{r}-b\left(c+s_{2}+t-dc\right)+\gamma \left(c+s_{1}+t-dc\right)\right]}^{2}}{16b}>0$.

Remark 9: The main difference between centralised decision-making mode and decentralised decision-making mode is that in centralised decision-making, the demand for direct sales channels will go down, but the demand for retail channels will start to rise. This will lead to a huge rise in the total demand of the supply chain, which will help manufacturers increase their overall export volume. From the perspective of the whole, under the centralized decision-making mode of the supply chain, compared with the decentralized decision-making mode, the profit yield can produce a significant gap, which is higher than the decentralized decision-making. This shows that the total return of the supply chain under the centralized decision-making model is much higher than that under the decentralized decision-making.

Remark 8 and 9 show that centralized decision making is beneficial to the whole supply chain and will optimize the optimal decision of the supply chain. For the supply chain, centralized decision-making not only increases the export volume and product market share of manufacturers, but also increases the profits of the entire supply chain. However, the effects of centralized decision making on manufacturing and retail entities have not been thoroughly studied, and the wholesale price of products by manufacturing entities has not been determined, so Remark 10 and 11 can be further deduced based on the above.

**Remark 10:** 1) when $\text{W}\geq \text{A},\pi_{\text{m}}^{C}-\pi_{m}\geq 0$; when $\text{W}<A,\pi_{\text{m}}^{C}-\pi_{m}<0$; 2) $\text{W}\leq \text{B},\pi_{\text{r}}^{C}-\pi_{r}\geq 0$; when $\text{W}>B,\pi_{\text{r}}^{C}-\pi_{r}<0$.


$$\text{A}=\frac{b\left(b^{2}-\gamma^{2}\right)\left[3c\left(1-d\right)-t-s_{2}\right]-\gamma \left(b^{2}-\gamma^{2}\right)\left[c\left(1-d\right)+t+s_{2}\right]+2b\gamma \left(a\rho +\mu_{m}\right)+\left(b^{2}+\gamma^{2}\right)\left(a+\mu_{r}-a\rho \right)}{4\left(b^{3}-b\gamma^{2}\right)};$$

$$\text{B}=\frac{b\left(b^{2}-\gamma^{2}\right)\left[5c\left(1-d\right)-3t-3s_{2}\right]-\gamma \left(b^{2}-\gamma^{2}\right)\left[c\left(1-d\right)+t+s_{2}\right]+4b\gamma \left(a\rho +\mu_{m}\right)+\left(3b^{2}+\gamma^{2}\right)\left(a+\mu_{r}-a\rho \right)}{8\left(b^{3}-b\gamma^{2}\right)};$$

Remark 10 shows that when W≥A, although the total profit of the supply chain of centralized decision making is larger than that of decentralized decision making, the distribution of profit is a problem. Only when the wholesale price is greater than a certain value, centralized decision-making is beneficial to the manufacturer. That is, compared with decentralized decision-making, when the external environmental factors are roughly the same, the profits distributed to the manufacturer under centralized decision-making mode are much higher than that under decentralized decision-making mode, the manufacturer will choose to actively participate in centralized decision-making. When W<A, the centralized decision is disadvantageous to the manufacturer, and the manufacturer will not choose the centralized decision. When W≤B, only when the wholesale price is greater than a certain value, centralized decision-making is beneficial to retailers, that is, when centralized decision-making can be allocated to retailers with greater profits under the same circumstances compared with decentralized decision-making, retailers will actively choose centralized decision-making. When W>B, centralized decision making is disadvantageous to retailers, and retailers will not choose centralized decision-making.

**Remark 11:** *B* ≤ W ≤ *A*时, $\pi_{\text{m}}^{C}-\pi_{m}\geq 0,\pi_{\text{r}}^{C}-\pi_{r}\geq 0$.

Remark 11 shows that the value range of wholesale price is within [*B*, *A*]. After statistical analysis and calculation of relevant data, from the perspective of profit acquisition, under the centralized decision-making mode of supply chain, compared with the decentralized decision-making mode, both manufacturing and retail entities have significantly improved. Therefore, if a certain conditional basis is reached, both the manufacturer and the retailer will choose to participate in the centralized decision, because the centralized decision will benefit both parties.

## 5. Numerical examples

This section uses numerical examples to explore the effects of tariff, transport heterogeneity and export tax rebate on the optimal pricing of direct selling and retail dual-channel supply chains, the demand of dual-channel supply chains and the profits of supply chain members, and whether the centralized decision-making of manufacturers and retailers can coordinate the effects of tariff, transport heterogeneity and export tax rebate on supply chains.

### 5.1 The impact of taxes and export rebates on supply chain decision-making

This section uses numerical examples to explore the effects of tariffs and export tax rebates on the optimal pricing, demand and profit of supply chain members in both direct and retail dual channel supply chains. The specific parameters are as follows: *α* = 100; *b* = 1.5; *γ* = 0.5; *ρ* = 0.5; *c* = *¥*20; *s*_1_ = *¥*2; *s*_2_ = *¥*1; *μ*_*m*_ = 5; *μ*_*r*_ = 4; *t* ∈ [0, 10], *θ* ∈ [0, 0.13] (Demand is measured in number; Prices are settled in RMB).

#### 5.1.1 Supply chain decision-making under decentralized decision-making

**Fig 1:**
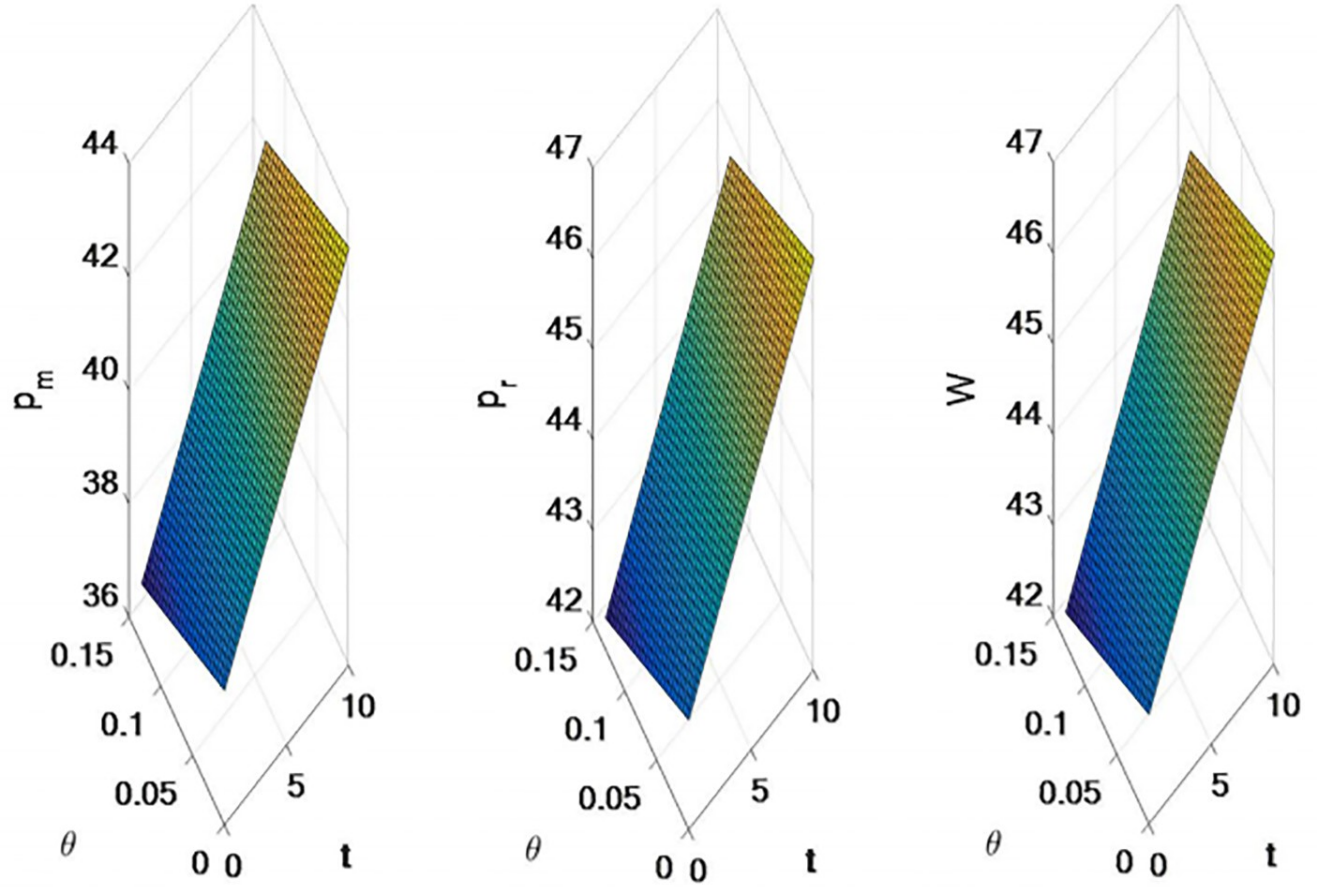
Effects of tariffs and export rebates on supply chain sales prices. Note: This distribution represents price differences under different conditions. *p*_*m*_ refers to the distribution of direct selling prices under the influence of tariff and export rebate conditions; *p*_*r*_ refers to the distribution of retail prices under the influence of tariff and export rebate conditions. *w* is the wholesale price distribution based on different decision models.

The results shown in Fig 1 are as follow:

1. with the increase of tariff, the sales price of online direct sales channel and traditional retail channel will increase.
2. the sales price of the online direct sales channel, the traditional retail channel sales price and the wholesale price will decrease based on the increase of export tax rebate rate. Export tax rebates will reduce the selling price of the entire supply chain.

#### 5.1.2 The impact of tariffs and export tax rebates on supply chain demand

**Fig 2:**
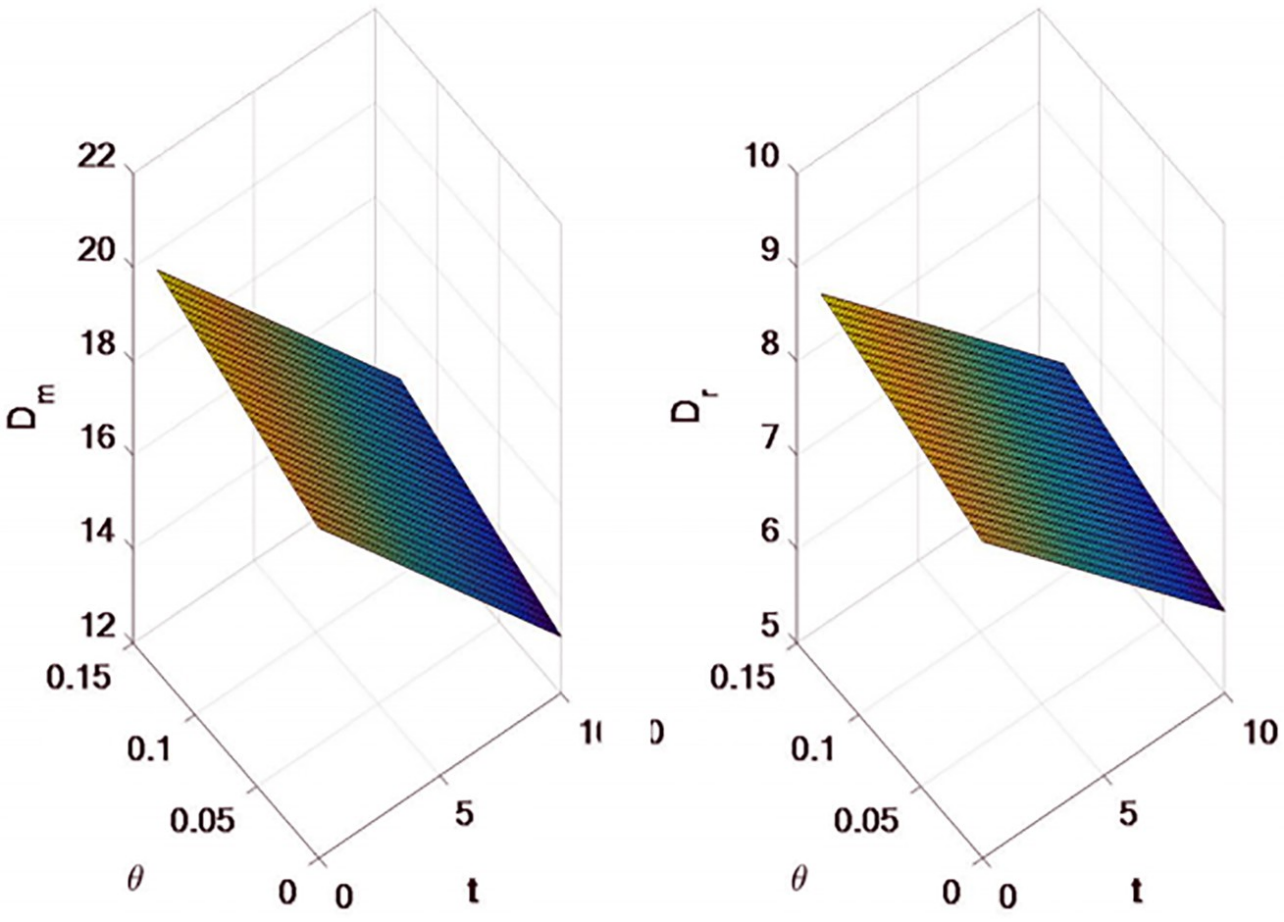
Effects of tariffs and export rebates on supply chain demand.

The results shown in Fig 2 are as follow:

1. with the increase of tariff, the demand for online direct selling channels and traditional retail channels will decrease accordingly, because the impact of tariff increase will eventually be transferred to consumers along the supply chain, that is, whether online direct selling channels or traditional sales channels, the purchase cost of consumers will increase, so the overall demand will decrease.
2. the demand of online direct selling channels and traditional retail channels will change based on the change of export tax rebate rate, which is reflected in the increase of export tax rebate rate, and the demand will also increase. Export tax rebates will increase the demand of the entire supply chain.

#### 5.1.3 The impact of tariffs and export tax rebates on supply chain profits

**Fig 3:**
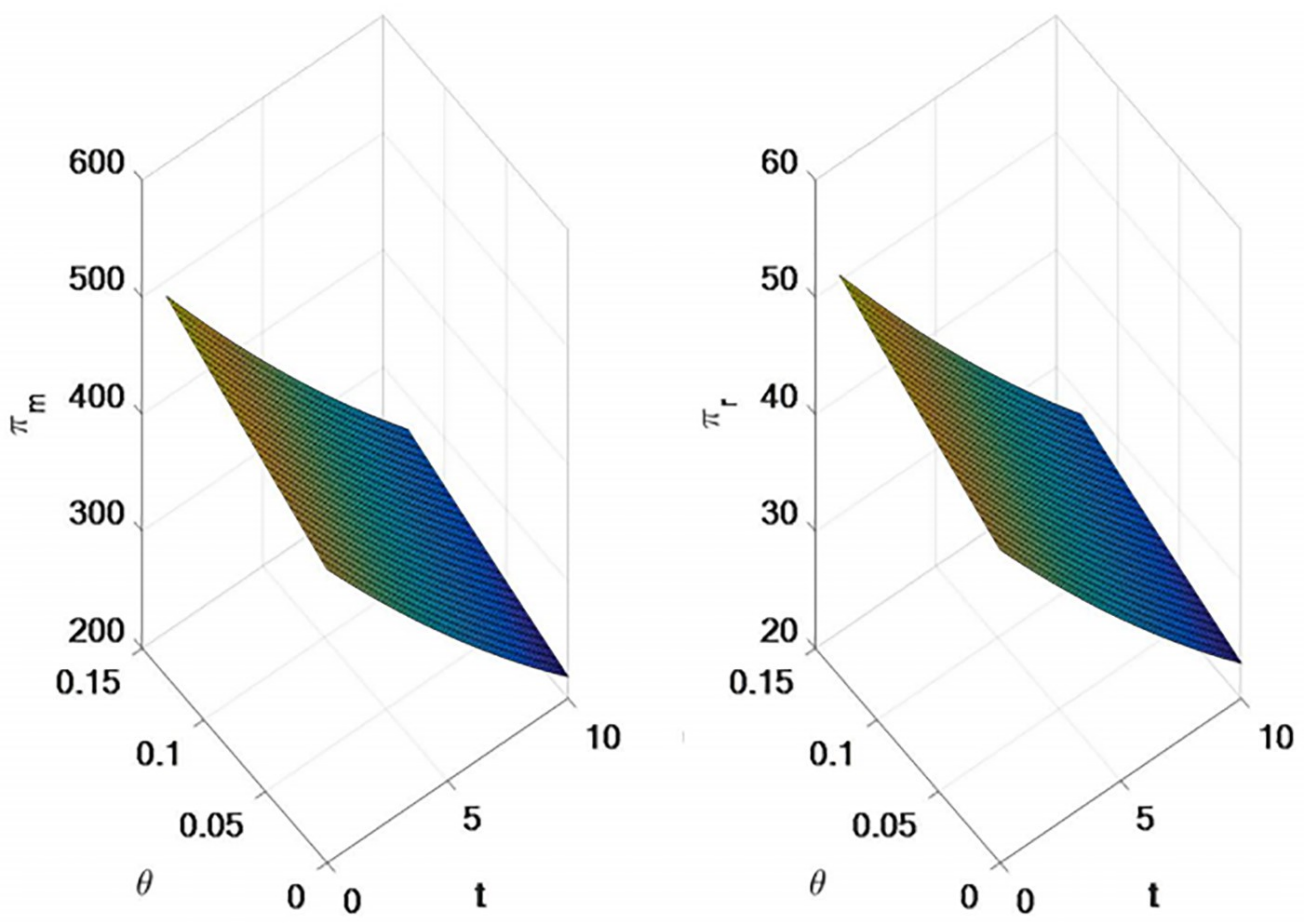
The effect of tariffs and export rebates on the profits of manufacturers and retailers.

The results shown in Fig 3 are as follow:

1. With the increase of tariffs, there is an inverse relationship between the profits of both manufacturers and retailers, and the profits begin to decrease.
2. The profit of manufacturers and retailers will change based on the change of export tax rebate rate, which shows that the increase of export tax rebate rate will increase the profit. Export tax rebates will increase the profits of the entire supply chain.

### 5.2 The influence of direct channel freight and retail channel freight on supply chain decision-making

In this section, the effects of transportation heterogeneity on optimal pricing, demand and supply chain member profit of dual-channel supply chain are discussed with numerical examples. Set related parameters as follows: *α* = 100, *b* = 1.5, *γ* = 0.5, *ρ* = 0.5, *c* = *¥*20, *μ*_*m*_ = 5, *μ*_*r*_ = 4, *t* = 5, *θ* = 0.13, *s*_1_ = [0, 5], *s*_2_ = [0, 5]. (Demand is measured in number; Prices are settled in RMB).

**Fig 4:**
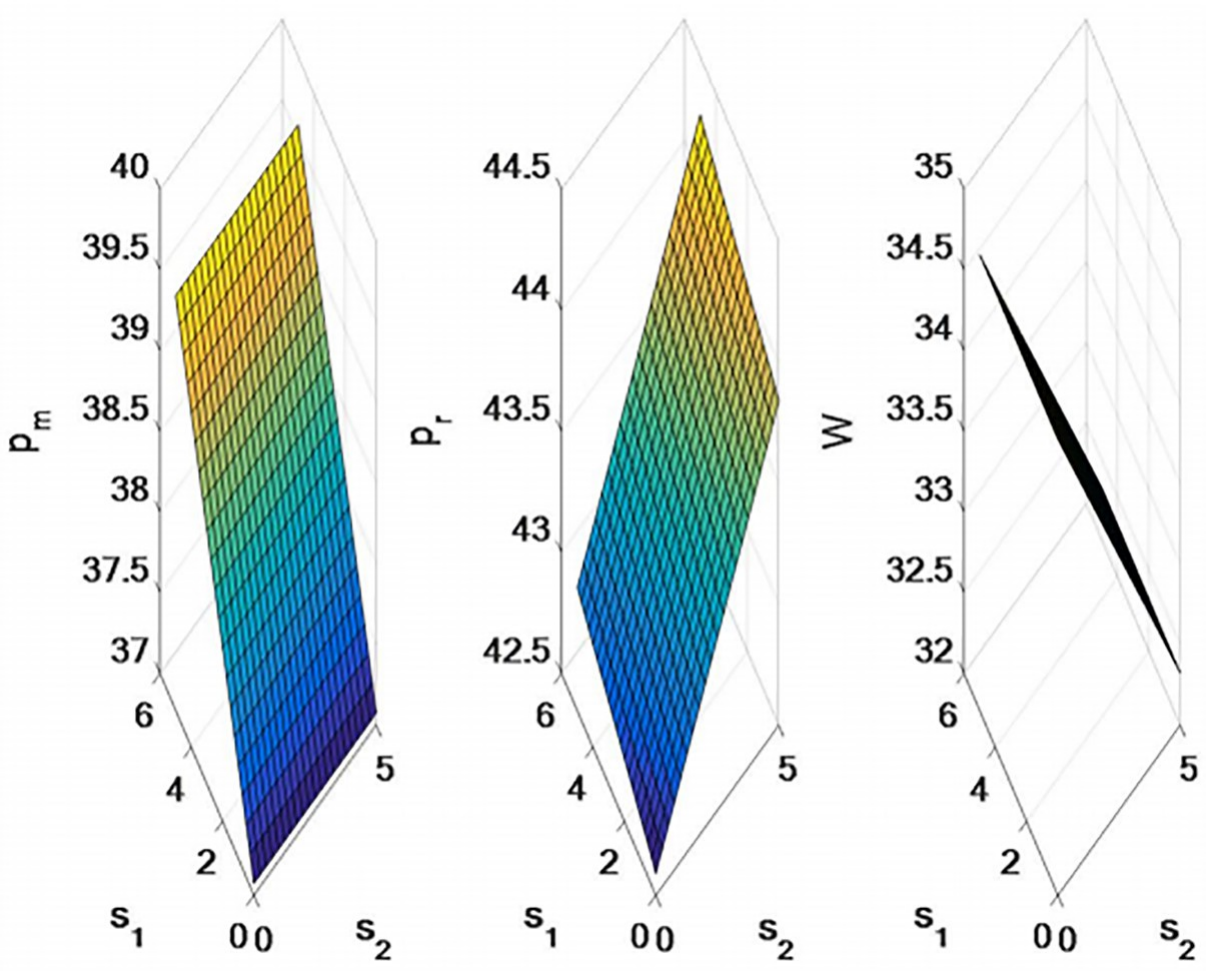
Impact of direct channel freight and retail channel freight on supply chain pricing.

As shown in Fig 4, with the increase of the unit freight of the direct selling channel, the direct selling price and retail price will increase, while the wholesale price does not change. As the retail channel unit freight increases, the wholesale price will decrease, the retail price will increase, and the direct sales price will not change.

**Fig 5:**
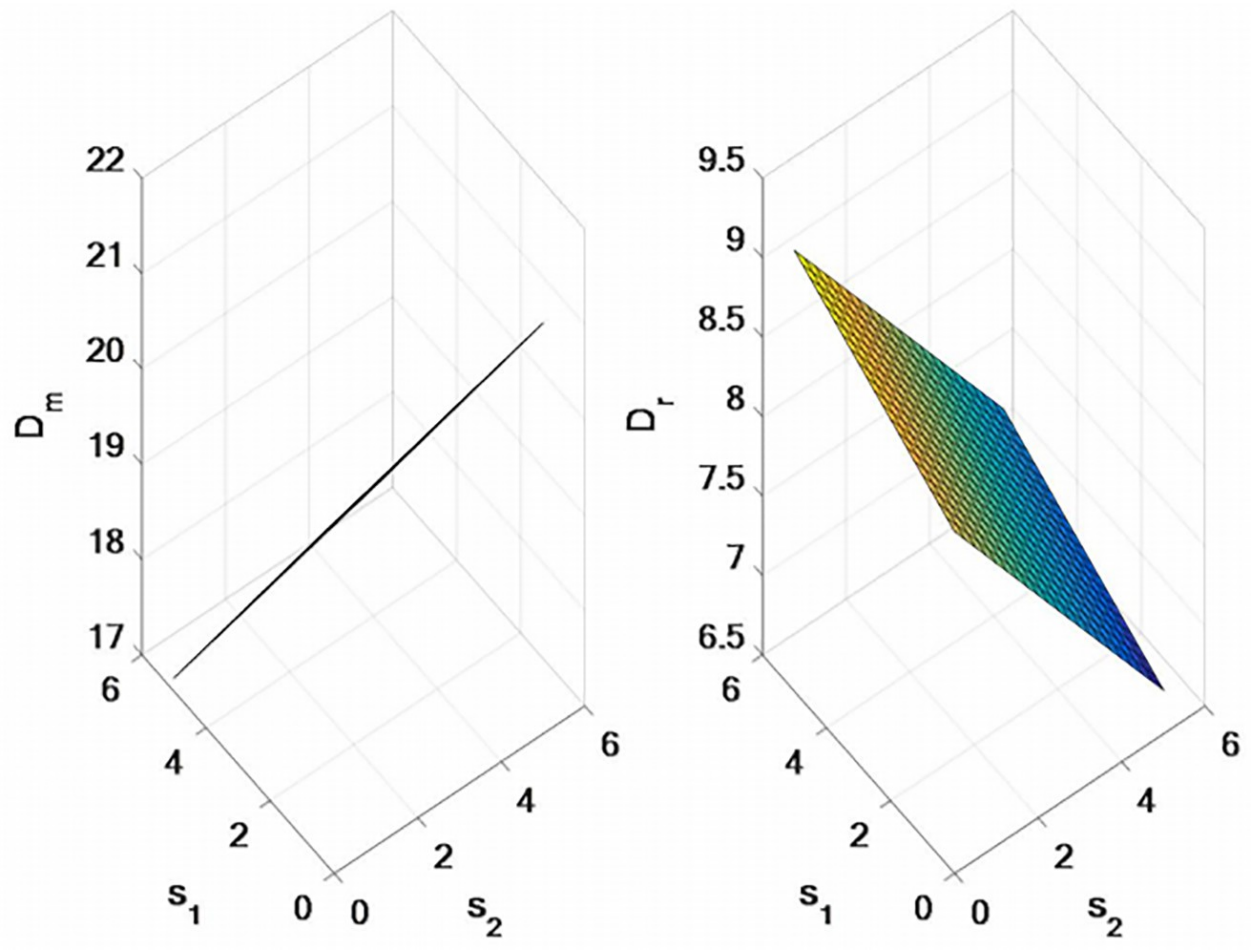
Impact of direct channel freight and retail channel freight on supply chain demand.

As shown in Fig 5, the demand of direct selling channel is negatively correlated with the unit cost and freight of direct selling channel, and positively correlated with the unit freight of retailer channel.

**Fig 6:**
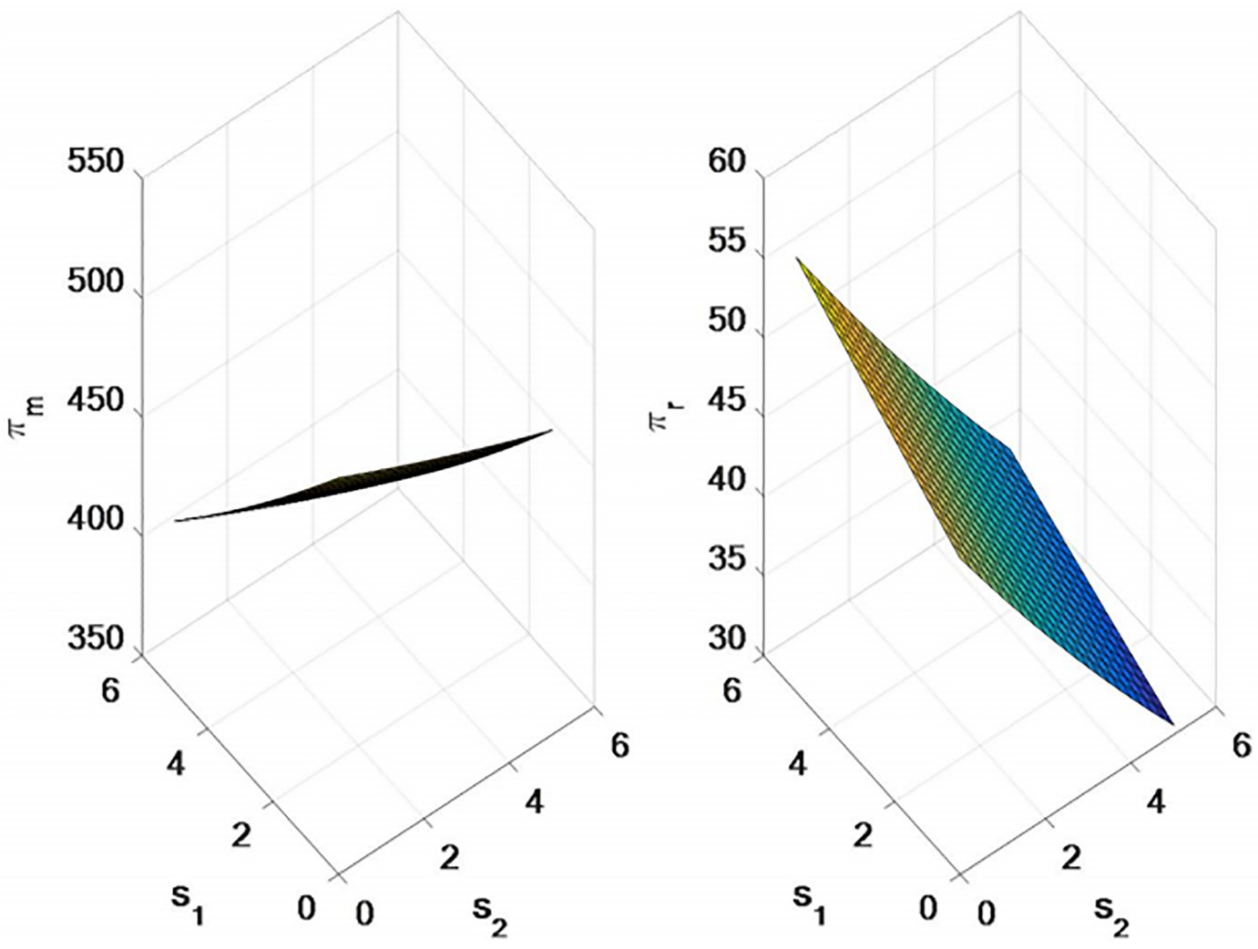
Impact of direct channel freight and retail channel freight on supply chain profits.

As can be seen from Fig 6, 1) Manufacturer’s profit will decrease with the increase of unit freight of retail channel and decrease with the increase of unit freight of direct selling channel. 2) Manufacturer’s profit will decrease with the increase of retail channel unit freight, and increase with the increase of direct channel unit freight.

### 5.3 Influence of centralized decision-making on supply chain decision-making

Based on numerical examples, this section studies and analyzes the effects of tariffs and export tariffs on the demand of the two sales channels (direct sales and retail) and the price of products at the time of sale under centralized and decentralized decision-making, and studies and analyzes the effects on the profits of manufacturing and retail entities under the same conditions.

*α* = 100, *b* = 1.5, *γ* = 0.5, *ρ* = 0.5, *c* = *¥*20, *s*_1_ = *¥*2, *s*_2_ = *¥*1, *μ*_*m*_ = 5, *μ*_*r*_ = 4, *t* ∈ [0, 10], *θ* ∈ [0, 0.13]. (Demand is measured in number; Prices are settled in RMB).

**Fig 7:**
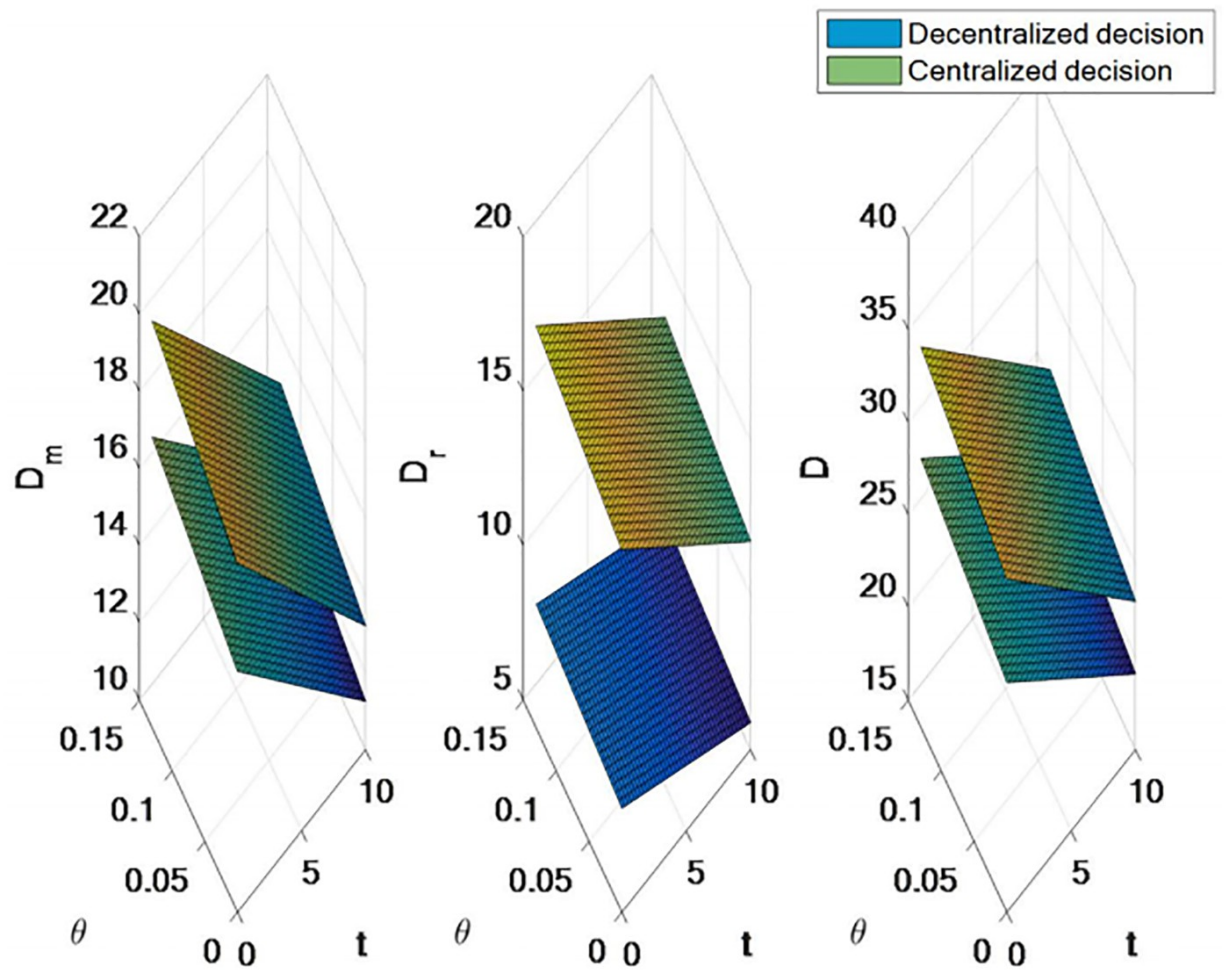
Comparison of requirements for centralized decision making and decentralized decision making.

As shown in Fig 7, compared with decentralized decision-making, centralized decision-making leads to a decrease in the demand for direct sales channels, a substantial increase in the demand for retail channels, and a great increase in the total demand of the supply chain. Therefore, it can be argued that centralized decision making is generally superior to decentralized decision making.

**Fig 8:**
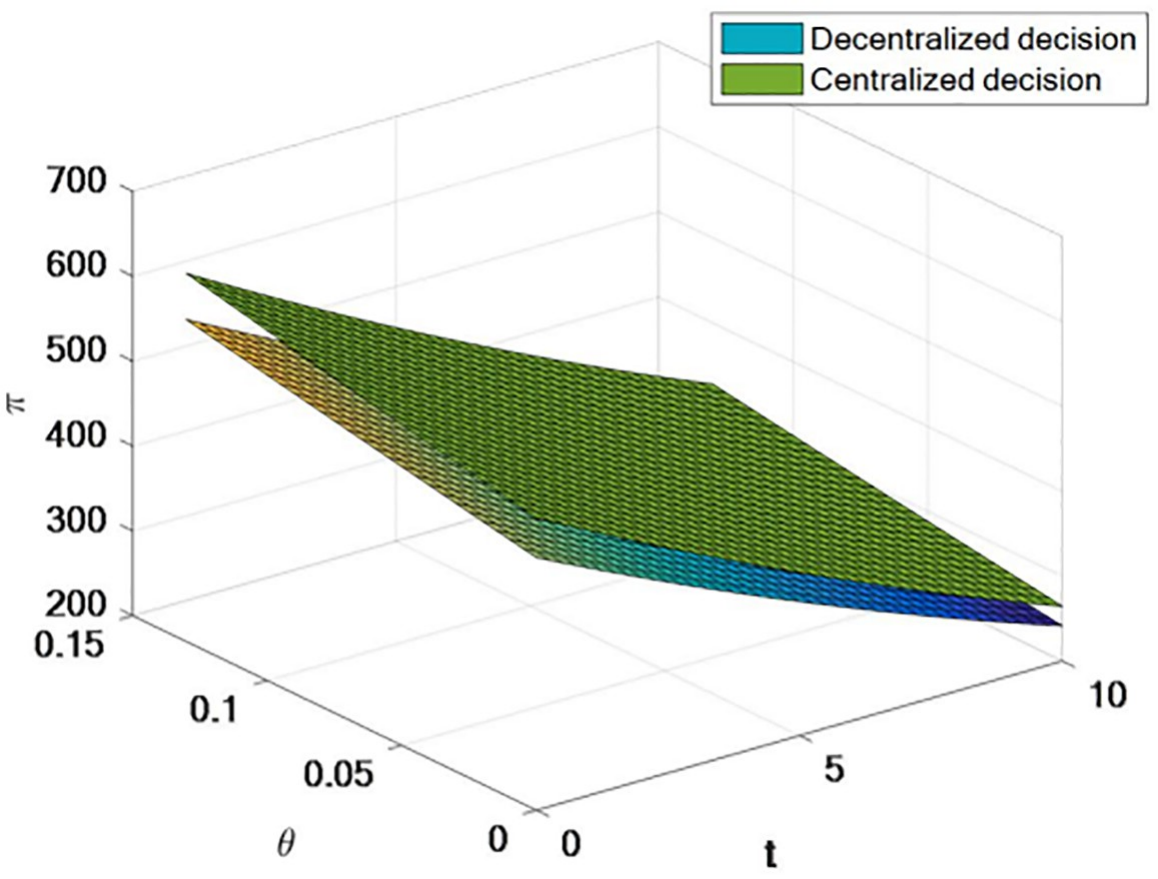
The influence of centralized decision making on supply chain profit.

Fig 8 shows that, for decentralized decision making, centralized decision making is more likely to increase the profits of the entire supply chain. Regardless of decentralized or centralized decision-making, the total profit of the supply chain is negatively correlated with tariffs and positively correlated with export tax rebates.

Table 3 demonstrates that the profit distribution of the entire supply chain can be influenced by the wholesale price, which is established collectively through both decentralised and centralised decision making. Enhancing the price qualities of products in a centralised decision-making mode can significantly boost producers’ profits, leading to an upward trend. Conversely, it will adversely affect retailers’ earnings, resulting in a downward trend. Furthermore, at W = 27.64, the earnings of manufacturers employing decentralised decision-making and centralised decision-making are equivalent. At W = 30.23, the retailer’s profit is approximately the same under both centralised and decentralised decision-making modes. The revenues of both manufacturers and retailers are considerably greater in centralised decision-making than in decentralised decision-making when the range of W is [27.64,30.23]. While centralised decision making can enhance the overall profitability of the supply chain, it is crucial to share the benefits in a sensible manner to avoid any negative impact on manufacturing and retail organisations. Manufacturing and retail businesses will choose for centralised decision-making within a specific range in order to obtain a mutually beneficial outcome.

**Table 3:** Centralized decision making is based on wholesale price allocation.

*wholesale price*	*Decentralized decision*			Centralized decision		
	*π*_*m*_	*π*_*r*_	*π*	*π*_*m*_	*π*_*r*_	*π*
*w* = 25.0000	387.6417	40.3004	427.9421	346.5638	121.6787	468.2425
*w* = 27.6417	387.6417	40.3004	427.9421	387.6417	80.6008	468.2425
*w* = 29.0000	387.6417	40.3004	427.9421	408.7638	59.4787	468.2425
*w* = 30.2333	387.6417	40.3004	427.9421	427.9421	40.3004	468.2425
*w* = 31.0000	387.6417	40.3004	427.9421	439.8637	28.3788	468.2425

## 6. Conclusion

Based on the background of cross-border dual-channel supply chain, the objective of this paper is to analyze the strategies of manufacturers and retailers under the conditions of tariff, export tax refund and transportation heterogeneity. By constructing relevant theoretical models, the paper conducts calculation research while considering two different decision-making modes of decentralization and concentration, and compares and analyzes the influence of optimal pricing, demand and member profit in the supply chain. In this paper, by constructing a model, some strategies are explained in numerical and deductive ways, so as to show the management views of the two main bodies in the current supply chain. As follows:

1. From the perspective of the overall benefits of the cross-border e-commerce dual-channel supply chain, manufacturers and retailers within the supply chain can effectively increase the profit income of the supply chain under the centralized decision-making mode.
2. From the perspective of domestic manufacturers, manufacturers should actively pay attention to import tariff policies and export products to countries with lower import tariffs; Manufacturers should actively fight for the export tax rebate policy and get a higher tax rebate rate. Manufacturers should find ways to reduce unit freight rates in direct channels, such as establishing inventory sharing mechanisms with retailers, and the retailer will ship.
3. From the perspective of foreign retailers, retailers should strive for lower tariff policies; Retailers should strive to reduce retail channel unit freight rates.
4. From the perspective of the government, low tariff and export tax rebate policies adopted by the government will increase manufacturers’ exports, which will benefit the entire supply chain.

Through the above analysis, it is beneficial for manufacturers and retailers to understand and adapt to the tariff policy and export tax rebate mechanism between different countries, and help them develop flexible logistics plans, help reduce costs, optimize production and sales strategies, and improve profit margins. Moreover, the model also enables manufacturers and retailers to simulate the effects of different decision-making patterns on various parties in the supply chain. These analyses and strategies are of great significance in academic research, and can directly guide practical business decisions to help enterprises cope with complex cross-border environments.

Although this paper mentions the role of factors such as tariffs, export rebates, and transportation heterogeneity in cross-border e-commerce supply chains, there are other important factors such as exchange rates in current practice in this area. Exchange rate fluctuations will directly affect the pricing strategies of cross-border e-commerce products in different channels. Usually, when the local currency appreciates, retailers will adjust the pricing of online channels to maintain the competitiveness of the international market, and adopt different pricing strategies for offline channels to maintain the attractiveness of local consumers. In addition, in the dual-channel operation, the exchange rate risk will be more complex, and the transnational e-commerce supply chain may adopt different exchange rate hedging strategies to maintain its own profit level. In addition, there are differences and randomness in the behavior patterns of consumers in different channels. There are obvious differences in the motivations and brand loyalty of different consumers in online channels and retail channels. And not only different individual consumers, but also different countries and regions will reflect different competitive environments and pricing strategies. Therefore, many different choices will be made based on supply chain decisions under various complex situations, and the model cannot be fully displayed due to the situation, which also reflects the potential limitations of the modeling assumptions in this paper.

The theoretical model proposed in this paper is based on realistic assumptions. According to the development stage of the supply chain in reality and the model design pointed out in this paper, further research objectives can consider the heterogeneity of consumers. Through the optimization of pricing and marketing strategies of the cross-border e-commerce supply chain, it is conducive to revealing the influence of the combination of different consumer groups and different channels, and improving sales efficiency and profit acquisition. Secondly, government policies in terms of tax, tariff preference and direct subsidies can significantly affect the cost structure and international competitiveness of manufacturers and retailers, and further affect the efficiency and market share of supply chain operations. Future studies can evaluate the actual impact of these policies on corporate decision-making and global market dynamics through empirical analysis. Provide more effective advice and strategic guidance to policy makers and businesses. By collecting and analyzing a large amount of actual data, the applicability and predictive ability of existing supply chain models can be verified under different market conditions. Such data-based verification helps to reveal possible limitations in existing models and provide more accurate and reliable supply chain strategy guidance for future research.

## Supporting information

S1 Data (DOCX)
