# Supplementary material for: Tariffs, transportation, and profits in cross-border e-commerce: A dual-channel supply chain decision-making strategy
Source: PLoS One. 2025 Jan 8;20(1):e0309535. doi: 10.1371/journal.pone.0309535 (PMC11709283; doi:10.1371/journal.pone.0309535)
Supplement: S1 Data — (DOCX) [file pone.0309535.s001.docx]

Chapter 4 Model solving

% The code for calculating retail price p2 in Section 4.1（Proposition 1 and remark 1-remark 6）

syms a b c d Q p s u q w r c g j n h t p cf q1 q2 u2 u1 s1 s2 v p1 p2 d2 d1 x

d1=p*a-b*(1+t)*p1+v*p2+u1

d2=(1-p)*a-b*p2+v*(1+t)*p1+u2

z1=(p1-(1-d)*c)*d1+(w-(1-d)*c)*d2

z2=(p2-(1+t)*w)*d2

diff(z2,p2)

solve(diff(z2,p2),p2)

p2=(u2 - a*(p - 1) + b*w*(t + 1) + p1*v*(t + 1))/(2*b)

% Calculation codes for wholesale price w and supplier price P1 in Section 4.1（remark 1-remark 6）

syms a b c d Q p s u q w r c g j n h t p cf q1 q2 u2 u1 s1 s2 v p1 p2 d2 d1 x

p2=(u2 - a*(p - 1) + b*w*(t + 1) + p1*v*(t + 1))/(2*b)

d1=p*a-b*(1+t)*p1+v*p2+u1

d2=(1-p)*a-b*p2+v*(1+t)*p1+u2

z1=(p1-(1-d)*c)*d1+(w-(1-d)*c)*d2

z2=(p2-(1+t)*w)*d2

diff(z1,p1)

diff(z1,w)

[solp1,solw]=solve('u2/2 - (a*(p - 1))/2 - (b*w*(t + 1))/2 + (p1*v*(t + 1))/2 - (b*(w + c*(d - 1))*(t + 1))/2 + (v*(p1 + c*(d - 1))*(t + 1))/2=0','u1 + a*p - (p1 + c*(d - 1))*(b*(t + 1) - (v^2*(t + 1))/(2*b)) - b*p1*(t + 1) + (v*(w + c*(d - 1))*(t + 1))/2 + (v*(u2 - a*(p - 1) + b*w*(t + 1) + p1*v*(t + 1)))/(2*b)=0','p1,w')

solutions=[solp1,solw]

% Computational code for centralized decision making in Section 4.2（Proposition 2 and remark 7-remark 11）

syms a b c d Q p s u q w r c g j n h t p cf q1 q2 u2 u1 s1 s2 v p1 p2 d2 d1 x

d1=p*a-b*p1+v*p2+u1

d2=(1-p)*a-b*p2+v*p1+u2

z1=(p1-s1-t-(1-x)*c)*d1+(w-(1-x)*c)*d2

z2=(p2-w-t-s2)*d2

z=z1+z2

diff(z,p1)

diff(z,p2)

[solp1,solp2]=solve('u2 - b*p2 - b*(w + c*(x - 1)) + p1*v + b*(s2 - p2 + t + w) - a*(p - 1) + v*(p1 - s1 - t + c*(x - 1))=0','u1 + a*p - b*p1 + p2*v + v*(w + c*(x - 1)) - v*(s2 - p2 + t + w) - b*(p1 - s1 - t + c*(x - 1))=0','p1,p2')

solutions=[solp1,solp2]

Chapter 5 Numerical examples

% Code for Sections 5.1 and 5.2

a=100

b=1.5

v=0.5

p=0.5

c=20

s1=2

s2=1

u1=5

u2=4

t=5

x=0.13

p1=(a.*v + b.*u1 + u2.*v + b.^2.*c + b.^2.*s1 + b.^2.*t - c.*v.^2 - s1.*v.^2 - t.*v.^2 + c.*v.^2.*x + a.*b.*p - a.*p.*v - b.^2.*c.*x)./(2.*(b.^2 - v.^2))

w=(a.*b + b.*u2 + u1.*v + b.^2.*c - b.^2.*s2 - b.^2.*t - c.*v.^2 + s2.*v.^2 + t.*v.^2 + c.*v.^2.*x - a.*b.*p + a.*p.*v - b.^2.*c.*x)./(2.*(b.^2 - v.^2))

p2=(u2 + p1.*v + b.*(s2 + t + w) - a.*(p - 1))./(2.*b)

d1=p.*a-b.*p1+v.*p2+u1

d2=(1-p).*a-b.*p2+v.*p1+u2

z1=(p1-s1-t-(1-x).*c).*d1+(w-(1-x).*c).*d2

z2=(p2-w-t-s2).*d2

z=z1+z2

D=d1+d2

surf(t,x,z);xlabel({'t'});ylabel('\theta ');zlabel({'\pi'});

hold on

subplot(1,3,2),surf(t,x,d2);xlabel({'t'});ylabel('\theta ');zlabel({'D_r'});

hold on

subplot(1,3,3),surf(t,x,D);xlabel({'t'});ylabel('\theta');zlabel({'D'});

hold on

% Code from Section 5.3

syms w

a=100

b=1.5

v=0.5

p=0.5

c=20

s1=2

s2=1

u1=5

u2=4

t=5

x=0.13

w=31.00

p1=(a.*v + b.*u1 + u2.*v + b.^2.*c + b.^2.*s1 + b.^2.*t - c.*v.^2 - s1.*v.^2 - t.*v.^2 + c.*v.^2.*x + a.*b.*p - a.*p.*v - b.^2.*c.*x)./(2.*(b.^2 - v.^2))

p2=(a.*b + b.*u2 + u1.*v + b.^2.*c + b.^2.*s2 + b.^2.*t - c.*v.^2 - s2.*v.^2 - t.*v.^2 + c.*v.^2.*x - a.*b.*p + a.*p.*v - b.^2.*c.*x)./(2.*(b.^2 - v.^2))

d1=p.*a-b.*p1+v.*p2+u1

d2=(1-p).*a-b.*p2+v.*p1+u2

z1=(p1-s1-t-(1-x).*c).*d1+(w-(1-x).*c).*d2

z2=(p2-w-t-s2).*d2

z=z1+z2

D=d1+d2

surf(t,x,z);xlabel({'t'});ylabel('\theta ');zlabel({'\pi'});

hold on

subplot(1,3,2),surf(t,x,d2);xlabel({'t'});ylabel('\theta ');zlabel({'D_r'});

hold on

subplot(1,3,3),surf(t,x,D);xlabel({'t'});ylabel('\theta');zlabel({'D'});

hold on
